# Ten-year anemia as initial manifestation of Castleman disease in the abdominal cavity: A case report

**DOI:** 10.1515/biol-2022-0898

**Published:** 2024-06-27

**Authors:** Pingping Xiao, Qingqing Wang, Zhigao Dong, Junnan Su, Yongquan Chen, Wei Fan

**Affiliations:** Department of Hematology and Rheumatology, The Second Affiliated Hospital of Xiamen Medical College, Xiamen, P.R. China

**Keywords:** Castleman disease, anemia, case report

## Abstract

Castleman disease (CD) is a relatively rare lymphoproliferative disorder. Lesions predominantly originate on the chest and neck and rarely occur on the abdomen. A 34-year-old female presented to our hospital with an unexplained 10-year history of anemia. A pathological diagnosis of plasma cell-type CD was established. One cycle of chemotherapy (thalidomide, cyclophosphamide, and prednisolone) improved her anemia significantly. Prompt etiological diagnosis and early intervention are essential to address systemic manifestations in patients with CD, and it is crucial to consider CD as a differential diagnosis when intra-abdominal masses are detected.

## 1 Background

Castleman disease (CD), a rare, non-neoplastic, lymphoproliferative disorder first described by Castleman et al. in the 1950s, is characterized by localized lymph node hyperplasia. CD is clinically classified into unicentric and multicentric CD (UCD and MCD, respectively) based on the number of regions of enlarged lymph nodes. The diagnosis is based on the pathology of the biopsy mass, with three pathological subtypes identified: hyaline vasculature (HV), plasma cells (PC), and mixed subtypes. The incidence of CD is less than 1 per 100,000, with UCD found to be more predominant in females than in males (2:1 ratio) [1]. Notably, 80–90% of UCD are pathologically HV, whereas 10–20% are PC CD [2]. The most common systemic symptoms include fever, anemia, night sweats, fatigue, and weight loss [3]. UCD has a good prognosis and a 5-year survival rate exceeding 90% and no impact on long-term survival outcomes [4]. Here, we report a patient who experienced unexplained anemia for 10 years that had remained undiagnosed. A lesion observed in the abdominal mass was diagnosed as plasma cell-type UCD (PC-UCD). Anemia improved after one cycle of chemotherapy, resulting in patient recovery.

## 2 Case presentation

A 34-year-old female presenting with a 10-year history of palpitations and breathlessness was referred to our hospital. Upon physical examination, her findings were as follows: blood pressure, 111/74 mmHg; heart rate, 96 beats/min; and respiratory rate, 19 breaths/min. She did not present with superficial lymphadenopathy, splenomegaly, hepatomegaly, or sternal tenderness and lacked any relevant personal or familial medical history.

Her laboratory blood test results were as follows: hemoglobin (HGB) level, 49.0 g/L (normal range [NR], 110–150 g/L); white blood cell count, 3.86 × 10^9^/L (NR, 4–10 × 10^9^/L); platelet count, 343 × 10^9^/L (NR, 100–300 × 10^9^/L); serum iron, 2.73 µmol/L (NR, 9–27 µmol/L); serum ferritin, 124.90 ng/mL (NR, 11.0–306.8 ng/mL); total iron-binding capacity, 31.92 µmol/L (NR, 45–75 µmol/L); transferrin saturation, 8.55%; serum folate, >53.47 nmol/L (NR, 11.8–50.0 nmol/L); serum B12, 160.63 pmol/L(NR, 180–914 pmol/L); reticulocyte percentage, 1.95% (NR, 0.3–3.0%); serum glutamic oxaloacetic transaminase, 8 IU/L (NR, 13.0–35.0 IU/L); serum glutamic pyruvic transaminase, 4 IU/L (NR, 7.0–40.0 IU/L); serum γ-glutamyl transpeptidase, 30 IU/L (NR, 7.0–45.0 IU/L); serum lactate dehydrogenase, 66.55 IU/L (NR, 100.0–240.0 IU/L). Serum alkaline phosphatase level was elevated (250 IU/L; NR, 35–100 IU/L). Creatine kinase and creatine kinase isoenzyme levels were within the NR. The erythrocyte sedimentation rate (ESR) was 154 mm/h (NR, 0–20 mm/h). Direct and indirect antiglobulin test results were negative, the immunoglobulin A level was 8.20 g/L (NR, 0.70–4.00 g/L), and the immunoglobulin G level was 30.60 g/L (NR, 7.00–16.00 g/L). The results of antinuclear antibody, anti-extractable nuclear antibody, antiphospholipid antibody, antineutrophil cytoplasmic antibody, rheumatoid factor, and anti-cyclic citrullinated peptide tests were negative. Tumor indices, including alpha-fetoprotein, carcinoembryonic antigen, CA199, and CA125, were within the NR. The erythropoietin level was elevated (70.27 mIU/mL; NR, 5.44–26.25 mIU/mL). Thyroid function was normal. The Epstein–Barr virus-DNA level was low (<4.0 × 10^2^ copies/mL). The IgG4 value was 788.9 mg/L (adult NR, 39.2–864 mg/L). There were no abnormalities in both α-thalassemia and β-thalassemia genes. Bone marrow morphology and biopsy revealed proliferative anemia. Flow cytometric analysis detected no developmental abnormalities in the bone marrow. Immunofixation electrophoresis of the serum and urine revealed polyclonal hypergammaglobulinemia. Bone marrow metaphase cytogenetics revealed a 46, XX karyotype.

**Figure 1:**
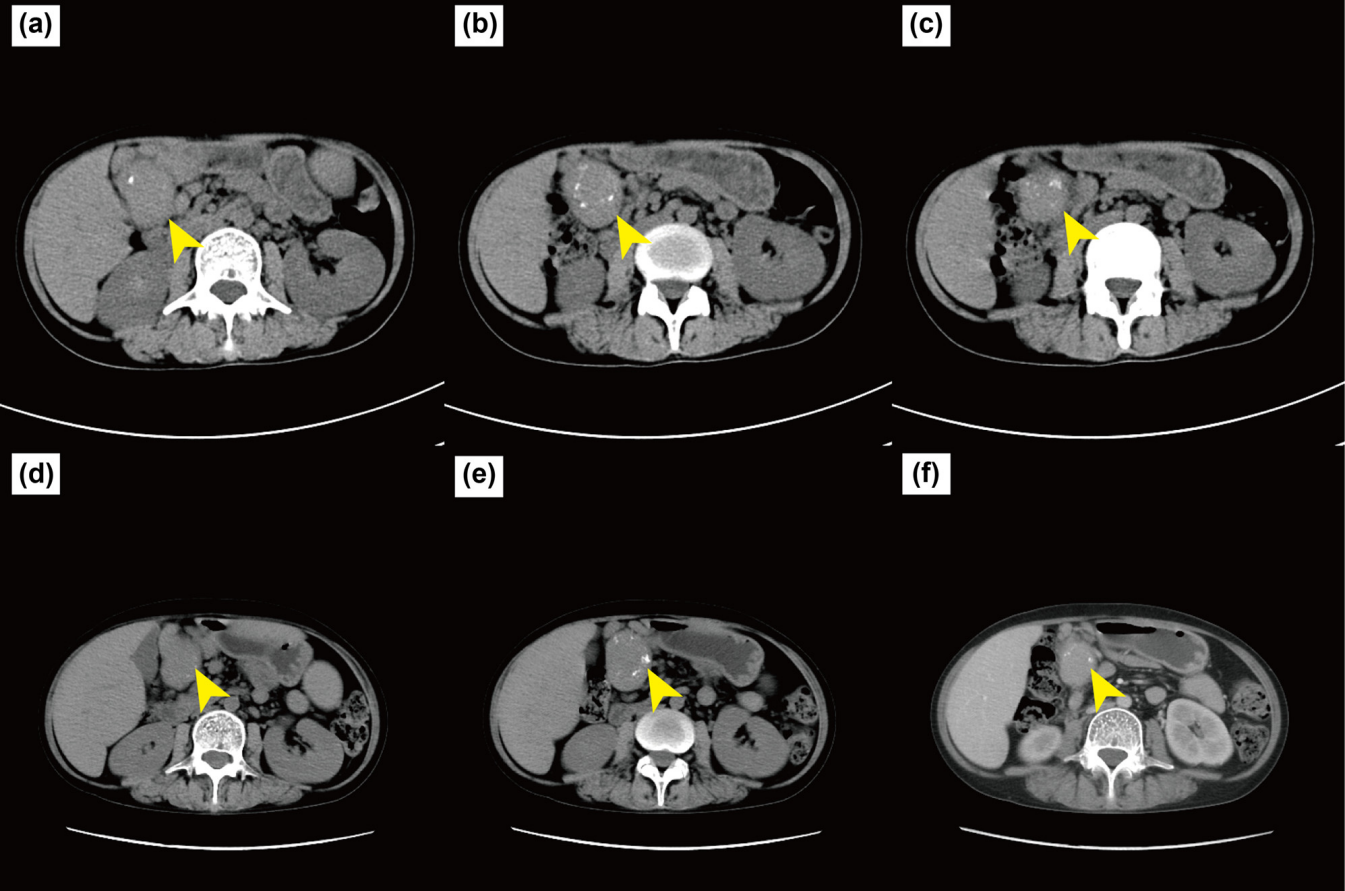
Abdominal computed tomography. Before (a)–(c) and after (d)–(f) chemotherapy axial; yellow arrow, upper left mass.

**Figure 2:**
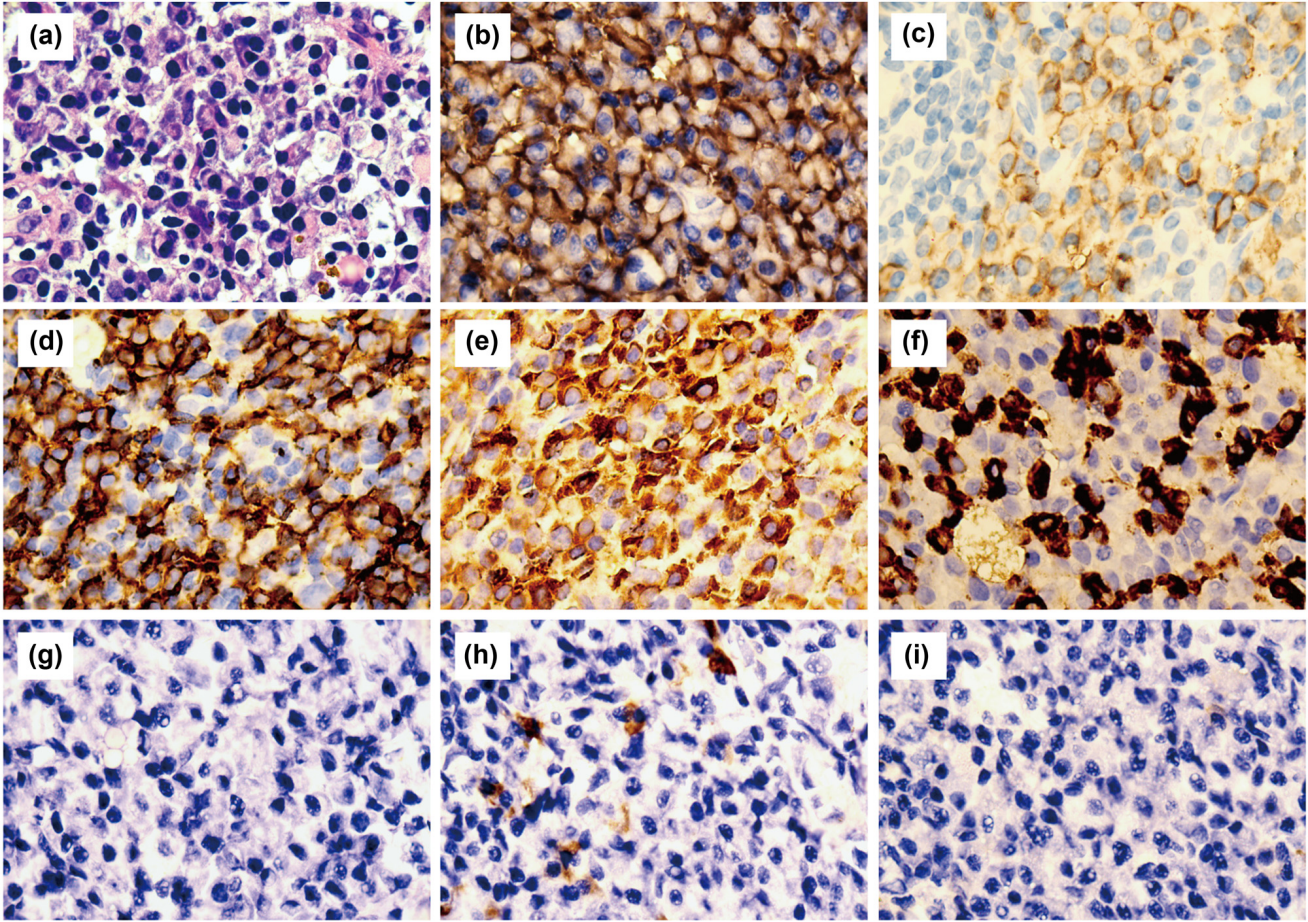
Abdominal cavity mass biopsy: (a) Hematoxylin & eosin staining ×1,000, (b) CD138-positive cells ×1,000, (c) CD38-positive cells ×1,000, (d) CD20-positive cells ×1,000, (e) Kappa-positive cells ×1,000, (f) Lambda-positive cells ×1,000, (g) HHV-8-negative cells ×1,000, (h) IgG 4-scattered positive cells (0–5 cells/HPF) ×1,000, and (i) CD56-negative cells, ×1,000.

At admission, computed tomography scans of the chest and whole abdomen were performed, revealing increased T4 and T11 bone density, a compression fracture, splenomegaly, a mid-abdominal mass, and peripheral lymphadenomegaly (Figure 1a–c). A punch biopsy of the abdominal cavity mass was performed under ultrasonographic guidance, and pathological tissues were diagnosed as lymphoid tissue hyperplasia with a significant increase in plasma cells; PC-CD was considered initially. Immunohistological analysis revealed the following findings: plasma cells with CD38, CD138, CD19, Kappa and Lambda positivity, HHV8-negative, CD56-negative, IgG4-scattered positive (approximately 0–5 pcs/HPF), B lymphocytes with CD19 and CD20 positivity, T lymphocytes with CD3 and CD5 positivity, Cyclin D1-negative, and Ki-67 (+5–10%) (Figure 2).

**Figure 3:**
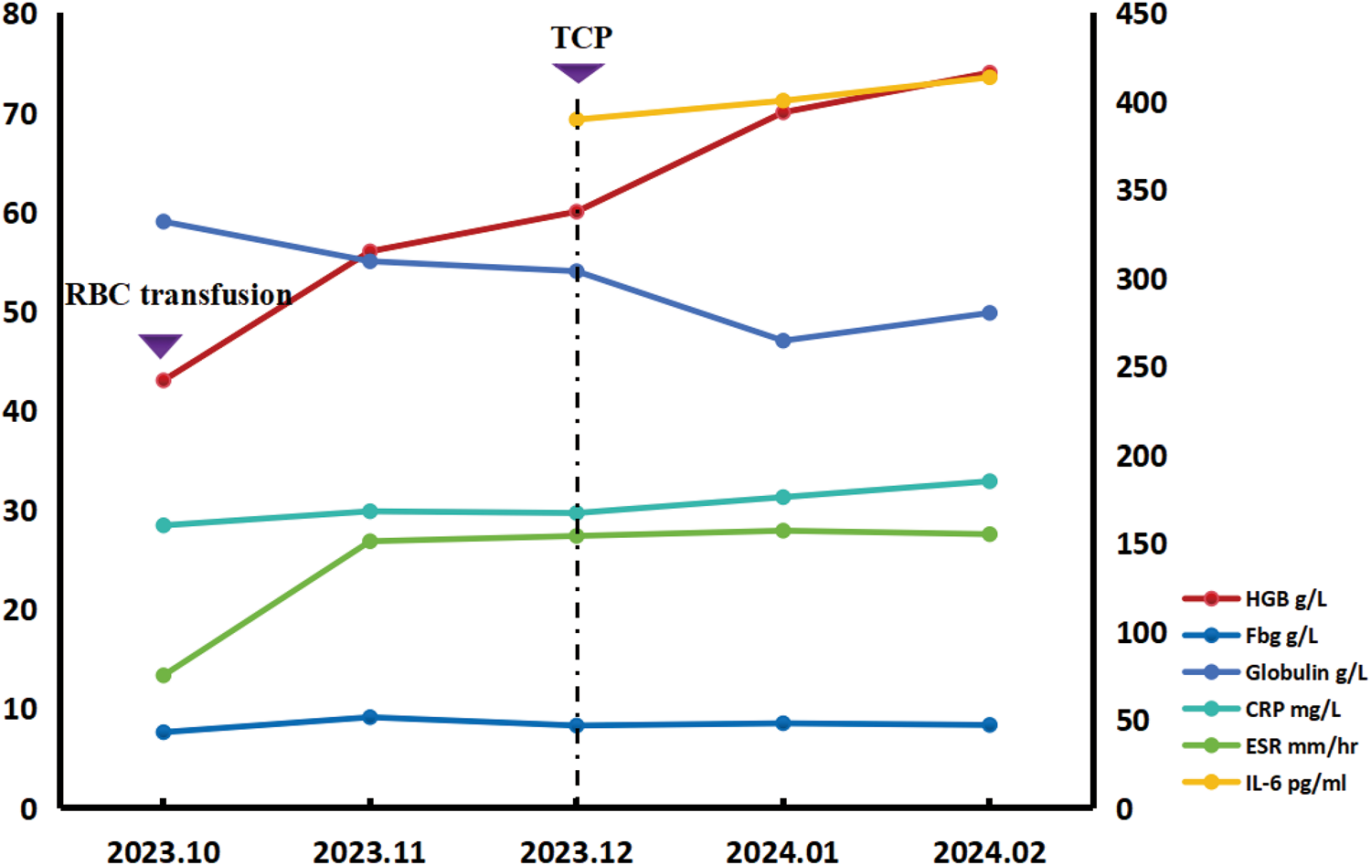
Changes in indices pre- and post-chemotherapy. HGB, hemoglobin; CRP, C-reactive protein; ESR, erythrocyte sedimentation rate; Fbg, fibrinogen; IL-6, interleukin-6; RBC, red blood cell; TCP, thalidomide, cyclophosphamide, prednisolone.


No clinically relevant or possibly pathogenic variants were identified using whole exome sequencing. Consequently, a diagnosis of PC-UCD was established. Following a multidisciplinary evaluation and communication with the patient, one cycle of chemotherapy (thalidomide, 75 mg/day; cyclophosphamide, 400 mg once a week; prednisolone, 40 mg twice a week) was administered. The size of the enlarged abdominal cavity mass did not decrease significantly (Figure 1d–f). However, her HGB levels gradually increased while her globulin level decreased. No reduction in C-reactive protein (CRP), ESR, fibrinogen (Fbg), and interleukin (IL)-6 levels were observed (Figure 3). The patient was able to engage in physical activities.

**Informed consent:** Informed consent has been obtained from all individuals included in this study.
**Ethical approval:** The research related to human use has been complied with all the relevant national regulations, institutional policies and in accordance with the tenets of the Helsinki Declaration, and has been approved by the authors’ institutional review board or equivalent committee.

## 3 Discussion

Herein, the patient had a 10-year history of unexplained anemia, with a significant reduction in her HGB level observed in the most recent 2 years. Anemia is more common in MCD and is rarely reported in UCD. Some cases of PC-UCD associated with iron deficiency anemia have been reported [5–8]. However, our patient failed to meet the diagnostic criteria for iron deficiency anemia in the previous 10 years. Oksenhendler et al. reviewed 57 patients with UCD, including 13 patients with an original mass in the abdominal cavity and only one patient with anemia [9]. Additionally, Patel et al. reported 791 adult cases, among whom 376 patients had anemia; however, the authors did not differentiate between UCD and MCD [10]. According to the current literature, the UCD type is associated with a low incidence of anemia, especially severe anemia. Our patient had experienced severe anemia for 10 years, during which she had fainted due to anemia on several occasions and required emergency transport to a hospital by ambulance.

We focused on related indicators, including ESR, CRP, Fbg, and IL-6 values, with higher values of these indices observed pre-chemotherapy (Figure 3). Subsequent blood analysis results revealed elevated HGB levels and reduced globulin, indicating improvement in her condition. However, her ESR, CRP, IL-6, and Fbg levels failed to improve significantly. CD is associated with elevated CRP and IL-6 levels [11]. In our patient, these levels had increased significantly prior to diagnosis and treatment. High levels of inflammatory indices after treatment, including ESR, CRP, IL-6, and Fbg, persisted, indicating the presence of substantial inflammation.

Compared with other factors such as ESR, CRP, and Fbg, IL-6 plays a pivotal role in hepcidin regulation in the pathogenic cascade that induces CD-related anemia; this cascade is initiated via IL-6-mediated hepcidin induction and subsequently results in anemia [12]. Genetic and molecular abnormalities in CD have been identified in novel subtype-specific pathways; for example, the over-representation of genes involved in collagenous fibrosis has been detected in UCD. Moreover, complement effector C3 and components of the classical component pathway (UCD: C1S and C1R) were found to be upregulated, suggesting that complement activation plays a role in the B-cell activation/plasma cell differentiation and the inflammatory response observed in UCD [13]. The mammalian target of rapamycin and Janus kinase signal transducer and activator of transcription pathways have been identified as potential therapeutic targets [14,15]. In UCD, severe clinical manifestations may be associated with molecular alterations in these pathways [16]. *FAS, PDGFRB, FGFR3, NF1*, and *TGFBR2* were identified as the most commonly affected genes involved in mitogen-activated protein kinase pathways in UCD [17]. Biomarkers *IL6ST, PDGFRB, DPP6*, and *MUC4* are the most frequently mutated genes in paraneoplastic pemphigus-associated UCD [18]. Moreover, chromosome abnormalities were found to involve chromosome 7 in UCD [19]. Our patient had a normal female karyotype (46, XX). Additionally, whole exome sequencing did not identify any related genes. Accordingly, the mechanism underlying anemia occurrence appears to be markedly complicated in PC-UCD and does not merely involve IL-6, which explains the improved anemia without a reduction in the persistently elevated IL-6 level post-treatment.

Therefore, optimal treatment options for our patient merit further investigation. Based on these findings, the next suggested line of treatment is either surgical excision of the mass or other chemotherapy regimens. Furthermore, we reviewed recent literature regarding the treatment of UCD. Talat et al. examined 278 patients with UCD, of whom 49 had abdominal cavity involvement and underwent surgery (97.1%). The authors reported a 10-year cumulative survival rate of >90% [20]. Boutboul et al. assessed 71 patients with UCD, 19 of whom had an abdominal cavity mass, and 11 had anemia. Of the 71 patients, 47 underwent surgery, of whom 43 (91%) achieved complete remission postoperatively. The estimated 5-year overall survival rate of 71 patients with UCD was 98.4% [21]. Guo et al. evaluated 118 patients with UCD, seven of whom underwent surgery as the primary treatment, achieving a 5-year overall survival of 88.1% [22]. Rodriguez et al. reported a 29-year-old female with a pelvic presentation of UCD, which was successfully treated with radiotherapy combined with chemotherapy regimens, thereby demonstrating promise in unresectable cases [23]. Surgery may be mutilating and associated with a high risk of complications. Radiotherapy and chemotherapy have been proposed to address unresectable tumors. Nevertheless, alternative options should be discussed. Our patient experienced poor general condition and could not tolerate surgical intervention. As outlined previously, our patient’s abdominal mass showed low Ki-67 expression. Ki-67, a cell proliferation marker, has been detected in several cancer types, including non-Hodgkin lymphomas, multiple myeloma, soft tissue sarcoma, prostate cancer, and breast cancer, with Ki-67 exhibiting prognostic value in predicting cancer survival rates and the likelihood of relapse [24]. Considering our patient, the abdominal mass with low Ki-67 expression (+5–10%) suggested low proliferative capacity. Consequently, we selected chemotherapy to improve anemia rather than initial surgical intervention. The treatment was the same as that for MCD. Prior to the UCD diagnosis, our patient had relied on recurrent red blood cell (RBC) transfusions. Post-diagnosis, the patient received one cycle of chemotherapy, which improved anemia and ceased the need for RBC transfusions. Currently, the patient undergoes regular follow-up, with elective surgery planned in the near future. Based on the findings in our case, one limitation is that anemia needs to be improved with appropriate chemotherapy prior to undertaking surgery in patients with PC-UCD. To address systemic complications such as severe anemia, chemotherapy may be an alternative therapy.

## 4 Conclusion

Although most patients with UCD have long survival, systemic manifestations, such as fever and severe anemia, impact their quality of life. Early etiological diagnosis is essential for patients with combined systemic symptoms, such as severe anemia observed in our case. Prompt selective surgery or medical treatment is the first step following a comprehensive evaluation.
